# The Path for Men from Young Adulthood Results of Cognitive Tests to Subclinical Atherosclerosis at Age 60: The Mediating Role of Socioeconomic Status, Lifestyle and Cardiovascular Disease Risk Factors–Results from a VIPVIZA Study

**DOI:** 10.31083/RCM26312

**Published:** 2025-03-20

**Authors:** Margareta Norberg, Per Liv, Ulf Näslund, Per Wester, Elin M Andersson, Steven Nordin

**Affiliations:** ^1^Department of Public Health and Clinical Medicine, Umeå University, 901 87 Umeå, Sweden; ^2^Department of Psychology, Umeå University, 901 87 Umeå, Sweden

**Keywords:** cognitive ability, atherosclerosis, cardiovascular risk, lifestyle, socioeconomic status

## Abstract

**Background:**

The role of cognitive abilities in the development of arteriosclerotic disease is still not fully understood. The purpose of the present study was to evaluate the mediating role of lifestyle, socioeconomic status (SES) and conventional cardiovascular disease (CVD) risk factors in the association between cognitive ability at age 19 and subclinical atherosclerosis at age 60 years.

**Methods:**

An observational study design was employed. Data on the results from cognitive tests of conscripts tested at age 19 were collected for 1009 men. At the age of 60, they were included in the trial VIsualiZation of asymptomatic Atherosclerotic disease for optimum cardiovascular prevention, which was conducted as part of the Västerbotten Intervention Program (VIPVIZA). VIPVIZA is a randomised controlled trial, aimed at primary prevention of CVD in Västerbotten County, Sweden. Prior to any intervention, they underwent carotid ultrasonography and CVD risk factor assessment. Lifestyle habits and marital status were self-reported, and education and urban or rural residency were registered. Crude associations between cognitive ability at age 19 and the risk of CVD, assessed with the European Systematic Coronary Risk Evaluation 2 (SCORE2), as well as subclinical atherosclerosis, as demonstrated by the presence of carotid plaques (no plaque, plaque unilateral, or plaque bilateral), were evaluated. A path-analytic model tested mediating factors from cognitive ability in young adulthood to subclinical atherosclerosis at age 60.

**Results:**

Results from cognitive tests at age 19 were in separate unadjusted analyses inversely and linearly associated with SCORE2 and with subclinical atherosclerosis. The association with carotid plaque at age 60 was mainly indirect and mediated by adult SES, which in turn had its main effect through adherence to healthy lifestyle habits via CVD risk of carotid plaques.

**Conclusions:**

Cognitive ability at age 19 is a factor that is upstream of adult SES and our study indicates that cognitive ability at a young age has long-term consequences via SES and lifestyle habits for CVD risk and atherosclerosis.

**Clinical Trial Registration:**

NCT01849575, https://clinicaltrials.gov/study/NCT01849575?term=NCT01849575&rank=1.

## 1. Introduction

Cognitive ability or intelligence at a young age has been found to be inversely associated with the risk of cardiovascular disease (CVD) morbidity and mortality in several large cohorts, mainly of European [1, 2, 3, 4, 5] or US [6] origin and in reviews [7, 8, 9]. Evaluations have included direct effects of early life cognitive ability on adult CVD and indirect (mediating) effects via childhood and adult socioeconomic status (SES), health behaviours, biological CVD risk factors, and genetic traits. However, the causal link between cognitive ability at a young age and social inequity in CVD later in life, and the role of mediating factors are still not fully understood [9]. Thus, more knowledge is needed to shed light on the role of early life cognitive ability in the development of atherosclerotic disease and on its impact on CVD prevention measures. Thus, filling this knowledge gap may guide development of public health strategies and methods to tailor individual CVD prevention efforts aimed at reduction of social inequity in health.

Atherosclerotic disease is the underlying process in most CVD events. Subclinical atherosclerosis, i.e., among apparently healthy people, is particularly relevant for CVD prevention strategies, because its progress can be slowed or even reversed through pharmacological treatment [10] or lifestyle changes [11, 12]. In addition, noninvasive imaging methods can conveniently detect subclinical atherosclerosis [10]. Contrary to atherosclerosis, which slowly evolves over the course of a lifetime, general cognitive ability is substantially stable from a young age [9]. However, to the best of our knowledge, compared to associations between results from cognitive tests at young age and established CVD at an older age, corresponding associations with subclinical atherosclerosis have been sparsely evaluated and the evaluations have presented conflicting results. In a cross-sectional study from 2008 with male participants aged 66–75 years, a higher score on a verbal test was negatively correlated to having carotid stenosis greater than 30%, and this association was only minimally attenuated by childhood and adult SES and CVD risk factors. Also, a negative association with intima-media thickness (IMT) has been shown. This was strongly attenuated by SES and CVD risk factors [13]. In contrast, another study showed a negative association between childhood intelligence and atherosclerosis, as demonstrated by lower IMT determined by carotid ultrasonography at age 50 years, but the effect size was not diminished by adjustment for SES, health behaviours or biological CVD risk factors [14].

Overall, the methods for evaluation of the associations between young adulthood cognitive ability and late adulthood CVD morbidity and mortality have shifted. Earlier studies employed regression methods (e.g., Cox proportional hazard ratio, logistic or linear regression). The strength of the associations was tested through adjustment for confounding by socioeconomic, behavioural, and clinical risk factors. Such procedures might lead to overadjustment when confounders are related to both the exposure and the outcome [15]. Instead, techniques considering interaction variables and structures of covariance, path analyses or other types of structural equation modelling (SEM) have gained ground [9]. Despite this, the nature of the underlying mechanisms, and the degree to which SES, health behaviours, and clinical risk factors mediate, moderate or confound the associations between early life cognitive ability and subclinical atherosclerosis in late adulthood still need more clarification.

Given this background, we utilised a comprehensive dataset to shed light on the association between results of cognitive ability tests at age 19 and subclinical atherosclerosis 40 years later. We had two aims. First, to evaluate whether there were any crude associations between young adulthood cognitive ability and risk of CVD and atherosclerosis at the subclinical stage, as demonstrated by presence of carotid plaques, at age 60 years Second, if a crude association was present, our primary aim was to explore this association by testing a path-analytic model in which:

(a) cognitive ability has an impact on SES, adherence to a recommended healthy lifestyle, CVD risk and atherosclerosis;

(b) SES has an impact on adherence to lifestyle recommendations and CVD risk;

(c) lifestyle has an impact on CVD risk;

(d) CVD risk has an impact on atherosclerosis.

## 2. Materials and Methods

### 2.1 Design, Setting and Study Population

Västerbotten Intervention Program (VIPVIZA) is an on-going population-based Pragmatic Randomised Open Blinded End-point trial (PROBE) conducted in Västerbotten County, Sweden, and has previously been described in detail [16]. Participants were invited to the trial when they took part in the Västerbotten Intervention Program (VIP). In the VIP, primary health care centres since the early 1990s invite all county inhabitants the year they become 40, 50, and 60 years old to a health survey that include conventional CVD risk factor assessments and comprehensive questionnaires on demographics, health, health behaviours, demographics, psychosocial factors, quality of life, and medication. Based on the results, an individual motivational dialogue with a trained nurse is provided, aiming at prevention of CVD and diabetes [17, 18].

Inclusion criteria to VIPVIZA were (i) age 60 years, or (ii) family history of CVD before age 60 years among first-degree relatives, or (iii) age 50 years and at least one of the following CVD risk factors: smoking, diabetes, dyslipidaemia, abdominal obesity, or hypertension. These criteria were aimed at a selection of the study population with a larger than normal fraction of persons at low or intermediate CVD risk, which was motivated by the fact that most CVD events occur in this large group [19]. The VIPVIZA population was collected over the entire county (area of 1.3 times that of Switzerland). The baseline ultrasound examinations were performed between April 2013–June 2016. To reduce selection bias due to urban versus rural place of residency and SES, remote rural areas were also included. The recruitment goal, based on power calculations prior to the start of the trial, was met and 3532 participants were included. Participants were consecutively and randomly assigned to either an intervention or a control group. However, because this study only utilised baseline data that was collected before the VIPVIZA intervention was provided to the intervention group, it was an observational study and data from the whole male study population was used. A flow-chart over inclusion, baseline, and the one-year follow-up visits was previously provided [16]. To avoid differences due to inclusion criteria, only participants aged 60 years, born in the mid-1950s, were included in this study. Further, only men were included, because until 1980 women were not enlisted and tested for Swedish military service, which was the source for the data on cognitive ability at age 19 years.

### 2.2 Measures

#### 2.2.1 Cognitive Ability at Age 19 Years

For Swedish men born during the 1950s, testing of conscripts was mandatory at age ~19 years. Data from the Swedish Conscripts Register were used and cognitive ability at age 19 years was defined based on results from tests of four separate psychological abilities: logical-inductive ability (to understand and follow written instructions), verbal ability, visuospatial ability and technical understanding [20]. Results from each test were converted into normally distributed standard nine-point scales (stanine) with values from 1 to 9. It was previously shown that the four scales are strongly related, and cluster into one factor that is considered to represent general cognitive ability [9, 21]. As in previous research, we therefore used the mean of the four subtests as a measure of cognitive ability at age 19 years [5, 22].

#### 2.2.2 Risk of CVD

The risk of CVD was defined based on the European Systematic Coronary Risk Evaluation 2 (SCORE2) as the percentage of 10-year risk of fatal and non-fatal CV events. SCORE2 was based on systolic blood pressure, non-high density lipoprotein (non-HDL) cholesterol, smoking, age, and sex, and the values were obtained using published algorithms [23]. Data from the VIP visit was used. Blood pressure was measured with the person in a sitting position according to standardised clinical methods, and the mean of two measurements was recorded. Total cholesterol, low density lipoprotein (LDL)-cholesterol, and HDL-cholesterol were analysed at the nearest hospital with clinical methods. Non-HDL cholesterol was defined as the total cholesterol minus the LDL cholesterol. Smoking was self-reported (yes/no). According to guidelines, for those aged 60 years, a 10-year risk of <5% is considered a low risk, 5 to <10% a moderate risk and ≥10% a high risk [23].

#### 2.2.3 Subclinical Atherosclerosis

Subclinical atherosclerosis was measured at baseline in the primary prevention trial VIPVIZA before participants had experienced any symptoms. It was defined as the presence of plaques with three categories: no plaque, plaque on one side, and plaque on both sides. All participants were examined with carotid ultrasonography by a team of trained biomedical scientists according to a strict protocol with the same portable ultrasound system (Panasonic Health Care Station, Diagnostic Ultrasound System GM-72P00A, Panasonic Healthcare Corp., Newark, NY, USA). The carotid arteries on both sides were scanned, and the IMT was automatically measured at four predefined angles according to the Meyers arc [24]. The presence of carotid plaques was defined on both sides according to the Mannheim consensus during the examination session [25]. Sonographers were blinded to which group the person had been randomized to, and previous results. At the baseline examination, participants with stenosis of ≥50% of the carotid lumen were excluded from the study (n = 22 out of 4177 participants who were assessed for eligibility) [16].

#### 2.2.4 Lifestyle

Assessments of lifestyle were based on self-reporting in the questionnaires at the baseline VIP visit. Adherence to recommendations for health promoting physical activity, alcohol use, and eating habits were based on generally used definitions and were categorised into three levels, scored as 1, 2 or 3, where larger value was healthier. Physical activity was assessed using two questions on type of leisure time activities and time spent on physical activity, respectively, and the three categories were: sedentary, moderately intensive physical activity less than 150 minutes per week, and moderately intensive physical activity ≥150 minutes a week alternatively intensive physical activity ≥75 minute/week. The Alcohol Use Disorders Identification Test (AUDIT) score, range 0–40 points, was used for alcohol consumption. This score was arranged into three categories: ≥16 points = probable alcohol dependence, 8–15 points = risky alcohol consumption, ≤7 points = consumption not at risk. For diet, a Healthy Diet Score (HDS) was used, which reflected the level of the individual’s intake of foods and beverages in relation to guidelines from the Swedish Food Agency. The HDS was calculated by using the frequency of intake of eight food and beverage groups in the VIP Food Frequency Questionnaire and has been shown to be related to CVD risk factors [26]. HDS categories were established based on the tertiles, thus dividing the total VIPVIZA-population into thirds corresponding to the lowest, middle, and highest HDS scores. A lifestyle score that reflected the degree of adherence to these three recommended healthy behaviours was calculated as a sum scoring 3–9. Smoking was not included in this lifestyle score because it is included in SCORE2. Thus, smoking was considered a CVD risk factor on its own due to its direct effect on the cardiovascular system [23].

#### 2.2.5 Socioeconomic Status (SES)

Data from registers at Statistics Sweden was used for educational level and was grouped into three groups assigned 0–2 points based on highest attained education: compulsory level (≤9 years of education), high school (10–12 years), and college or university, including postgraduate education (≥13 years). Marital status was based on self-reporting and categorised as either living alone (= 0) or married or cohabiting (= 1). Residency was divided into rural (= 0) or urban (= 1), where the cities of Umeå and Skellefteå were considered urban and the rest of the county rural. The points were totalled into a score for SES of 0–4.

### 2.3 Statistical Methods

Characteristics are presented in percentage for categorical variables and median and interquartile range for continuous variables*. *

Aim 1: The crude association between cognitive ability at age 19 years and SCORE2 was analysed with linear regression analysis, where the logarithm of SCORE2 was used due to skewness. The regression coefficient representing the slope of the association on a log scale was retransformed using the exponential function and interpreted as a multiplicative factor for the association on the original scale. The crude association between cognitive ability at age 19 years and presence of plaque was estimated using a proportional odds model with plaque presence categorised as no plaque, unilateral plaque or bilateral plaque, as the outcome variable. Linearity was assumed between cognitive ability at age 19 years and the outcomes on a logarithmic scale. The linearity assumption was examined comparing the models with linear effects to corresponding models where cognitive ability at age 19 years was entered into the model using restricted cubic splines. Three knots were used, placed at the 10th, 50th, and the 90th percentile of the distribution, for cognitive ability at age 19 years. Comparisons between linear and non-linear associations were made using a likelihood ratio chi-square test for the proportional odds models and a general linear F-test for the linear regression models, as implemented in the function *anova*, in the R-package *rms*. Linear associations were not rejected for either of the two outcomes. Further, strata were formed by categorizing the SES and lifestyle indices at their medians. Interaction effects between SES strata and cognitive ability, and lifestyle index strata and cognitive ability, were examined in separate models for both SCORE2 and plaque. Regression analyses were conducted using R version 4.3.1 (R Core Team (2023). R: A Language and Environment for Statistical Computing. R Foundation for Statistical Computing, Vienna, Austria).

Aim 2: The anticipated model for subclinical atherosclerosis, described above under the aims, was tested with path analysis, which is a form of structural equation modelling for testing and estimating causal relationships by using a combination of statistical data and qualitative causal assumptions. As a first step, Spearman correlation coefficients were calculated between the variables in the model as a general description. A path analysis was then conducted, applying the maximum likelihood method. The root mean squared error of approximation (RMSEA) and Benler’s comparative fit index (CFI) were used as goodness-of-fit indices. An RMSEA value of 0.05 [27] and a CFI value of 0.95 [28] were indicative of a good model fit. The α-level was set at 0.05. The path analysis was performed using Statistical Package for the Social Sciences (SPSS), Analysis of Moment Structures (AMOS), version 28, IBM, Armonk, NY, USA.

## 3. Results

### 3.1 Study Population Characteristics

In VIPVIZA, 1054 men aged 60 years were included, and data on the results of cognitive tests at age 19 years was obtained for 1009 participants, who constituted the study population in this study. When comparing the 45 participants lacking data on cognitive ability at age 19 years with those with cognitive ability data, a larger proportion were living alone at age 60 years (42% versus 20%, *p* < 0.001), and more commonly living in urban areas (89% versus 57%). No other statistically significant differences were observed. The frequency of missing data in other variables was <1% for all variables, except for AUDIT, which was missing in 1.9% among all 1054 men.

Among the 1009 men, plaque was identified in 613 (60.8%). Regarding cognitive ability at age 19 years, all variables that were included in SCORE2, and variables on lifestyle and SES at age 60 years are presented by the plaque status in Table 1. Cognitive ability at age 19 years, SCORE2, systolic blood pressure, smoking, AUDIT score, and educational level differed significantly between the three groups (no plaque, plaque on one side, and bilateral plaque).

**Table 1.** **Cognitive ability at conscription testing age 19 years and characteristics at age 60 years by carotid plaque status in the study population**.

		Total	No plaque	Plaque on one side	Plaque on both sides	*p-*value
		N = 1009	N = 396	N = 276	N = 337	
Cognitive ability at age 19 years		4.20 (3.5, 5.00)	4.40 (3.60, 5.00)	4.20 (3.60, 5.00)	4.20 (3.30, 4.80)	0.006
SCORE2		7.28 (6.05, 9.19)	6.87 (5.87, 8.58)	7.25 (5.92, 9.05)	7.79 (6.34, 9.94)	<0.001
Systolic blood pressure, mmHg		131 (122, 140)	130 (120, 140)	131 (122, 140)	134 (124, 143)	0.002
Non-HDL cholesterol, mmol/L		4.14 (3.40, 4.92)	4.07 (3.40, 4.72)	4.27 (3.46, 4.94)	4.19 (3.32, 5.01)	0.255
Smoking, N (%)						0.029
	Daily smoking	79 (7.8)	21 (5.3)	21 (7.6)	37 (11.0)	
	Smoking, not daily	32 (3.2)	14 (3.5)	5 (1.8)	13 (3.9)	
	Non-smoker	896 (89.0)	361 (91.2)	250 (90.6)	285 (85.1)	
Physical activity, N (%)						0.138
	Sedentary	171 (17.0)	70 (17.7)	34 (12.4)	67 (20.1)	
	Moderate activity <150 minutes/week	289 (28.8)	116 (29.4)	79 (28.8)	94 (28.1)	
	Physically active ≥150 minutes/week	543 (54.1)	209 (52.9)	161 (58.8)	173 (51.8)	
AUDIT score		4 (2, 5)	3 (2, 5)	4 (2, 5)	4 (2, 5)	0.033
Healthy diet score		12 (10, 15)	13 (10, 15)	12 (10, 15)	13 (10, 15)	0.749
Education, N (%)						0.027
	Compulsory	159 (15.7)	56 (14.1)	36 (13.0)	66 (19.6)	
	High school	519 (51.4)	197 (49.7)	142 (51.4)	180 (53.4)	
	College/university	332 (32.9)	143 (36.1)	98 (35.5)	91 (27.0)	
Married or cohabiting, N (%)		799 (79.8)	318 (80.7)	220 (80.0)	261 (78.6)	0.779
Living in urban area, N (%)		572 (56.7)	237 (59.8)	159 (57.6)	176 (52.2)	0.109

Median and interquartile range is given for continuous variables and number and percentage for categorical variables. HDL, high density lipoprotein; SCORE2, European Systematic Coronary Risk Evaluation 2; AUDIT, Alcohol Use Disorders Identification Test.

### 3.2 Aim 1: Crude Associations

**Fig. 1.**
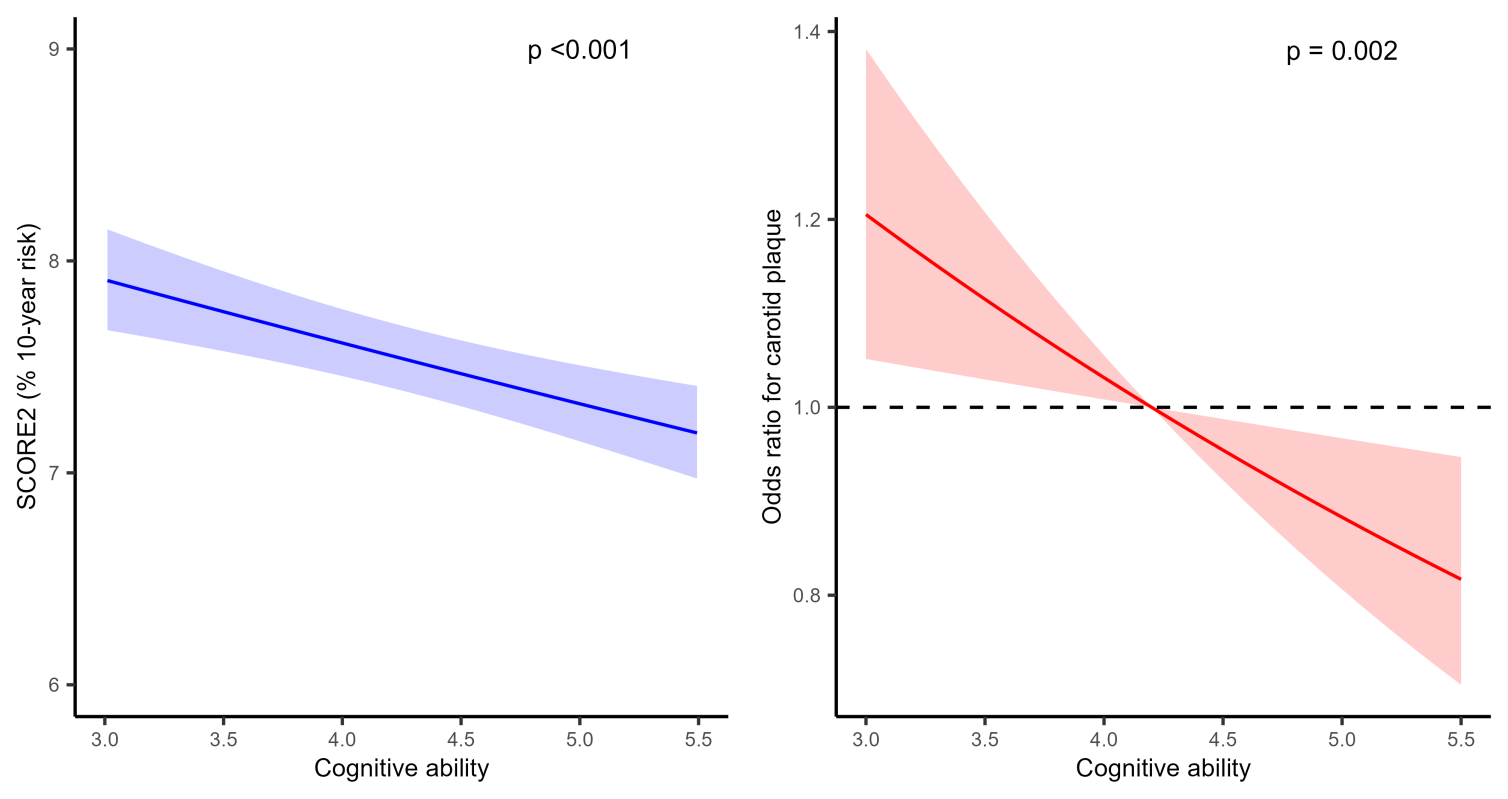
**Associations between cognitive ability at age 19 years and cardiovascular disease (CVD) risk (left) and presence of carotid plaque (right) at age 60 years**. The plaque variable includes three levels (no plaque, unilateral plaque, bilateral plaque). The reference for the odds ratio in the carotid plaque analysis is the median in cognitive ability at age 19 years of the study participants (4.2 points). SCORE2, European Systematic Coronary Risk Evaluation 2.

The crude association between cognitive ability at age 19 years, SCORE2 (left panel) and carotid plaque status (right panel) at age 60 years is shown with 95% confidence interval bands in Fig. 1. The exponentiated slope for this relationship was 0.96 (95% confidence interval (CI): [0.94, 0.98], *p <* 0.001), implying an expected 4% reduction of SCORE2 for each unit increase in cognitive ability at age 19 years. The odds ratio for having more carotid plaque on the three-level scale (no plaque, unilateral, bilateral) for a one unit increase in cognitive ability at age 19 years, as estimated from the proportional odds model, was 0.85 (95% CI: [0.77, 0.74]). There were no significant interactions between SES strata and cognitive ability on the effect on SCORE2 (*p* = 0.127) nor on plaque presence (0.153). The same results were seen for the interactions between lifestyle index strata and cognitive ability (*p* = 0.322 and 0.575, respectively).

### 3.3 Aim 2: Test of the Path-Analytic Model

**Fig. 2.**
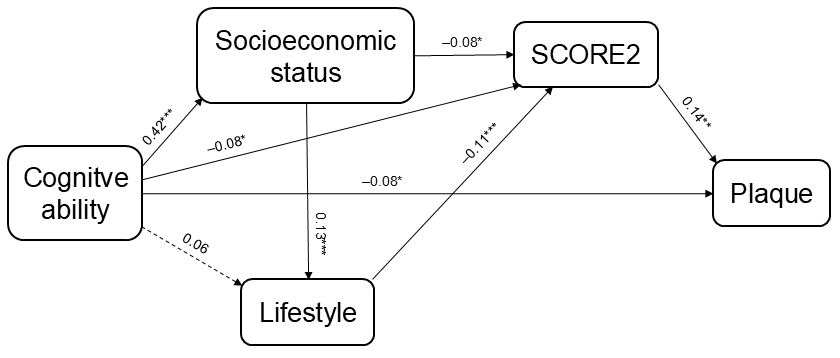
Results from the path-analytic model showing path coefficients (*p < 0.05, **p < 0.01, ***p < 0.001). The *p*-value for the association between cognitive ability at age 19 years and lifestyle was 0.065. Lifestyle included physical activity, diet and alcohol consumption. SCORE2 included smoking, systolic blood pressure, non-HDL cholesterol, age and sex. All variables except cognitive ability were measured at age 60 years. SCORE2, European Systematic Coronary Risk Evaluation 2; HDL, high density lipoprotein.

Correlations between the variables in the model are shown in Table 2. All five variables were significantly inter-correlated, except for the relationship between lifestyle index and presence of carotid plaque, which was not included in the model (Fig. 2). Measures of goodness-of-fit indices indicated a good fit (RMSEA <0.001; CFI >0.999). All path coefficients were significantly larger than zero, except for the association between cognitive ability at age 19 years and lifestyle (*p *= 0.065).

**Table 2.** **Spearman correlation coefficients between variables in the path-analytic model**.

	Socioeconomic status	Lifestyle	SCORE2	Plaque
Cognitive ability at age 19	0.402***	0.116***	–0.132***	–0.092**
Socioeconomic status		0.158***	–0.114***	–0.089**
Lifestyle			–0.112***	–0.027^ns^
SCORE2				0.143***

** *p* < 0.01, *** *p* < 0.001, ^ns^ non-significant. Lifestyle included physical activity, diet and alcohol consumption. SCORE2 included smoking, systolic blood pressure, non-HDL cholesterol, age and sex. All variables except cognitive ability were measured at age 60 years. SCORE2, European Systematic Coronary Risk Evaluation 2; HDL, high density lipoprotein.

## 4. Discussion

We found that cognitive ability at age 19 years is associated with subclinical carotid atherosclerosis at age 60 years. The results showed a linear inverse relationship between results of cognitive tests and subclinical atherosclerosis, defined as the presence of carotid plaque. This relationship was studied in detail by investigating potential mediating variables with path analysis. Although a path-analytic model is inherently causal by nature, causal interpretation of its results relies heavily on a correct specification of the model and other crucial non-verifiable assumptions, such as no unmeasured confounding factors. In the current setting, using the observational data at hand, a credible causal interpretation cannot be formed. However, the strongest path coefficient observed was from cognitive ability to SES, whereas variances explained by other predictor variables were statistically significant, but small. This indicates that the inverse association between cognitive ability at a young age and presence of plaque at age 60 years, observed in the crude analysis, was mainly mediated by adult SES, which, in turn, had its main effect on CVD risk through adherence to healthy behaviours (physical activity, diet, alcohol consumption), along with a small direct effect on the risk of CVD. Taken together, these factors led to a reduced risk of subclinical atherosclerosis. Our results concur with the review by Deary *et al*. [9] in which the links between intelligence at a young age and late life disease and death are outlined. They also match two other studies that focused on established CVD and utilising the testing data of Swedish conscripts. These two studies reported associations between low cognitive ability and a higher burden of CVD risk factors at hospital admission for a first myocardial infarction, and more non-adherence to statin treatment and lower two-year survival after discharge from the hospital [5, 22].

Our results may be interpreted considering health literacy. It was previously shown that low health literacy is associated with poor health, and that general cognitive ability and educational or occupational level account for most of this association [29]. Low health literacy is associated with inappropriate risk perception, feeling less capable to perform lifestyle changes, fewer proactive coping behaviours, and a greater likelihood of denying CVD [30]. This links to current clinical guidelines for primary prevention of CVD that address the importance of effective risk communication and assessing whether patients understand their risk, along with the pros and cons of an intervention [23]. Besides structural efforts to reduce social inequity in chronic diseases, CVD prevention strategies should also aim to bridge the inequity gap arising from differences in cognitive abilities. The use of imaging techniques has increased largely as diagnostic tools during recent years. They are also used to increase patients’ understanding and accuracy of risk perception. Reviews have found that communication strategies that provide visualized evidence to inform individuals about their actual atherosclerotic disease have the potential to promote primary preventive actions [31, 32]. However, more primary CVD prevention trials are warranted and they should be adequately powered and their intervention components and techniques described and evaluated in detail, in order to gain understanding of psychological mechanisms underlying successful interventions [31, 32]. These trials should use communication strategies that do not put strain on literacy, numeracy, and working memory functions, and instead communicate clear and easy-to-understand messages and contribute to empowering individuals. As illustrated by the linear inverse relationship between young age cognitive ability and subclinical atherosclerosis at age 60 years in this study, such strategies may be relevant for the vast majority of the general population, and not just for individuals at the lower end of the cognitive ability scale or with clear cognitive deficiency. Furthermore, early interventions targeting vulnerable groups are motivated because it has been suggested that there is a reciprocal relationship between executive functions and healthy behaviours [33]. From this perspective, further separate evaluations of the impact of the four lifestyle habits on the association of young adulthood cognitive ability and subclinical atherosclerosis at age 60 are warranted. However, these evaluations are beyond the scope of this report.

## 5. Limitations of the Study

The present study has both strengths and limitations. The strengths include a wide range of variables, the large sample size of healthy individuals, the VIPVIZA being integrated in ordinary healthcare including mainly people with a low to intermediate CVD risk, and the actual atherosclerotic disease, when demonstrated, being subclinical. These features enhance generalization to a relatively normal population rather than to a clinical CVD population. A limitation is the exclusion of women in the sample, restricting the results to be generalised only to men. Because the study population only includes men born in Sweden in the 1950s, this may limit the applicability of the findings to different generations, regions, or cultures. It is a limitation that area, composition, and characteristics of the plaques, which overall were small, were not evaluated in this subset from the VIPVIZA study population. Additionally, lifestyle was based on self-reported data, and mis-classifications could both attenuate and bias associations. To the best of our knowledge, the measures for cognitive abilities that were applied in the testing of conscripts during the 1960s and 1970s were general tests of fluid or crystallised intelligence. This means that certain important aspects of cognitive functioning, especially executive functions, were not assessed. It was beyond the scope of this study to evaluate the impact of determinants of cognitive ability at age 19 years on subclinical atherosclerosis at age 60, such as genetic factors and early life SES, as well as the impact of early life environmental circumstances. The cross-sectional design, even though the measurement of cognitive ability preceded the other variables in time, did not enable explicit tests of the model’s causal relations.

## 6. Conclusions

Our study indicates that results of cognitive tests at age 19 years have an impact on atherosclerosis at age 60 years, with SES being an important contributing factor influencing lifestyle. Hence, because cognitive ability at a young age is a factor that occurs before adulthood SES, focusing only on adulthood SES would be insufficient to reduce social inequity in CVDs.

## Data Availability

The dataset analysed during the current study is available from the PI of the VIPVIZA trial on reasonable request, patrik.wennberg@umu.se.

## Abbreviations

CVD, cardiovascular diseases; SES, socioeconomic status; VIPVIZA, VIsualiZation of asymptomatic Atherosclerotic disease for optimum cardiovascular prevention—a randomized controlled trial nested in the Västerbotten Intervention Program; VIP, Västerbotten Intervention Program; IMT, intima media thickness; SCORE2, the European Systematic Coronary Risk Evaluation 2; CI, confidence interval.

## Supplementary Material

Supplementary material associated with this article can be found, in the online version, at https://doi.org/10.31083/RCM26312.
